# Study of ExacTrac X‐ray 6D IGRT setup uncertainty for marker‐based prostate IMRT treatment

**DOI:** 10.1120/jacmp.v13i3.3757

**Published:** 2012-05-10

**Authors:** Chengyu Shi, Adam Tazi, Deborah Xiangdong Fang, Christopher Iannuzzi

**Affiliations:** ^1^ Department of Oncology St. Vincent's Medical Center Bridgeport CT USA

**Keywords:** ExacTrac, Novalis Tx, setup, marker, prostate, IMRT

## Abstract

Novalis Tx ExacTrac X‐ray system has the 6D adjustment ability for patient setup. Limited studies exist about the setup uncertainty with ExacTrac X‐ray system for IMRT prostate treatment with fiducial markers implanted. The purpose of this study is to investigate the marker‐based prostate IMRT treatment setup uncertainty using ExacTrac 6D IGRT ability for patient setup. Forty‐three patients with prostate cancers and markers implanted have been treated on the Novalis Tx machine. The ExacTrac X‐ray system has been used for the patient pretreatment setup and intratreatment verification. In total, the shifts data for 1261 fractions and 3504 correction times (the numbers of X‐ray images were taken from tube 1 and tube 2) have been analyzed. The setup uncertainty has been separated into uncertainties in 6D. Marker matching uncertainty was also analyzed. Correction frequency probability density function was plotted, and the radiation dose for imaging was calculated. The minimum, average, and maximum translation shifts were: −5.12±3.89 mm, 0.20±2.21 mm, and 6.07±4.44 mm, respectively, in the lateral direction; −6.80±3.21 mm, −1.09±2.21 mm, and 3.12±2.62 mm, respectively, in the longitudinal direction; and −7.33±3.46 mm, −0.93±2.70 mm, and 5.93±4.85 mm, respectively, in the vertical direction. The minimum, average, and maximum rotation shifts were: −1.23° ± 1.95°, −0.25° ± 1.30°, and −2.38° ± 2.91°, respectively, along lateral direction; −0.67° ± 0.91°, −0.10° ± 0.61°, and −1.51° ± 2.04°, respectively, along longitudinal direction; and −0.75° ± 1.01°, −0.02° ± 0.50°, and −0.82° ± 1.13°, respectively, along vertical direction. On average, each patient had three correction times during one fraction treatment. The radiation dose is about 3 mSv per fraction. With the ExacTrac 6D X‐ray system, the prostate IMRT treatment with marker implanted can achieve less than 2 mm setup uncertainty in translations, and less than 0.25° in rotations as overall interfraction mean error. The imaging dose is less than kV (CBCT) for setup verification.

PACS number: 87.55.km, 87.55.Qr, 87.56.bd

## I. INTRODUCTION

With the goal to achieve both treatment accuracy and precision for the intensity‐modulated radiotherapy (IMRT), the on‐board imaging system has played a very important role on the patient IMRT treatment.^(^ ^1^ ^)^ The Novalis Tx system (Varian Medical Systems, Palo Alto, CA) is a modality designed with the ability of image‐guided radiotherapy (IGRT) and IMRT. The integrated ExacTrac X‐ray 6D system (BrainLAB AG, Feldkirchen, Germany) has the ability for pretreatment positioning and intrafraction verification (snap mode). In the light of this system, for prostate patient IMRT treatment, it is possible to verify the target location before and intratreatment. With biomarkers implanted inside the prostate gland, it is more convenient for the ExacTrac X‐ray system to identify the location of the prostate and make correction for setup shifts. A tighter tolerance (1 mm) for isocenter can be set to limit the drifting range using snap mode. Any motion out of the tolerance can be adjusted during the treatment period. Therefore, both the treatment precision and accuracy can be justified.

Previous studies have reported the use of three IGRT modalities comparison among Varian OBI‐CBCT (Varian Medical Systems, Palo Alto, CA), BrainLAB ExacTrac and TomoTherapy MVCT (TomoTherapy Inc., Madison, WI) for prostate cancers treatment.^(^ ^2^ ^)^ However, intratreatment verification and adjustment was not reported. Tateoka et al.^(^ ^3^ ^)^ has done a study for patient setup for head and neck cancers treated using BrainLAB system, and showed the reproducibility was approximately ±1.0 mm and ±2.0 mm for translation errors and ± 1.2° and −1.0° to 3.0° for rotational errors for upper and lower region, respectively. The overall BrainLAB ExacTrac system performance for IGRT treatment has been reported within submillimeter range.^(^ ^4^ ^)^ The geometric accuracy of the 6D robotic couch system has been studied previously and it was found that the overall system accuracy was around 0.31±0.77 mm range.^(^ ^5^ ^,^ ^6^ ^)^ Intrafraction setup variability by X‐ray system for hypofractionated cranial and body radiotherapy has been investigated, and the motion range for abdomen was 1.3±1.2 mm ^(^ ^7^ ^)^ and 1.19±0.45 mm for the frame‐based technique.^(^ ^8^ ^)^

The latest designed Novalis Tx system has been used in clinical practice since 2008. However, limited studies^(^ ^9^ ^,^ ^10^ ^)^ exist for the application of IGRT/IMRT to prostate treatment with biomarker guidance and X‐ray 6D IGRT setup. In this study, we reported our results of setup uncertainty with the Novalis Tx ExacTrac X‐ray system for prostate IMRT treatment with fiducial markers implanted. The goals of this study are to answer the questions: How reliable were the therapists who did the setup alignment with markers?; What is the proper and robust tolerance for the prostate marker matching using ExacTrac X‐ray system?; and What is an adequate frequency to check the intrafraction motion?

## II. MATERIALS AND METHODS

### A. Treatment procedure

It is our clinical protocol that for every prostate patient IMRT treatment, we need biomarkers (either gold seeds or Visicoil (Core Oncology, Santa Barbara, CA)) implanted. The biomarkers can serve as landmarks for the patient pretreatment setup using the ExacTrac X‐ray system marker matching functionality. The 6D dynamic couch will move the patient to the treatment position after the markers have been registered. In order to account for the intrafraction motion effect, ExacTrac X‐ray snap mode has been used to verify the isocenter drifting with a tolerance of 1 mm. The patient will be asked to take some water about half an hour before the treatment and empty bowel if necessary, to keep full bladder and empty bowel. Tattoo markers will be used to align with the in‐room lasers for initial patient setup and infrapositioning array will be used to monitor the motion. X‐ray verifications will be done by acquiring patient X‐ray images from tube 1 and tube 2, and then the marker matching is done to calculate the 6D shifts. Once the shifts are applied, one more X‐ray verification image is taken. The patient will be advised to either fill the bladder or empty the bowel if the marker matching result shows large differences during the initial setup matching. During the treatment, snap mode will be used to monitor the patient intrafraction shift. If the snap verification is out of tolerance (> 1 mm), then the patient reposition will be applied. The snapshot of the treatment was usually taken in the middle of the treatment. If the isocenter shift was within the tolerance, the treatment was continued without correction. Otherwise, repositioning the patient was needed.

The treatment plan was developed using Eclipse treatment planning station (version 8.6, Varian Medical Systems, Inc., Palo Alto, CA). A clinical protocol was set up to develop a seven‐field sliding windows IMRT plan.

### B. Fiducial marker

We used Visicoil for about 80% of our patients and gold seeds for the remaining. Two coils or four gold seeds were usually implanted. Coil marker has the advantage of exhibiting less migration than the seed marker. However, both markers are working well so far, and can be easily identified on the ExacTrac X‐ray system.

### C. Statistics

In total, 43 patients had been treated before being randomly selected for this study. For the 43 patients, 1261 fractions and 3504 correction times in total have been analyzed. Here the correction times are the numbers of X‐ray images taken from tube 1 and tube 2. The selection of patients was based on the fully recorded reports for the whole treatment period. The recorded reports were exported from the BrainLAB ExacTrac console by way of the patient manager exporting the summary CVS file function. The reports should include all 6D shifting results and more than 1 fraction recorded. Each patient's files have been exported from ExacTrac system to a local drive. Setup shifts and markers coordinates were recorded in the exported files. The collected data was sorted by date, shift directions, and angles. The minimum, average, and maximum shifts have been calculated. The markers matching distances were also calculated in 3D. The correction frequency was summarized and plotted as probability density function (PDF). The final maximum, mean, and minimum values were averaged through the whole patient group after the values for each individual patient data were calculated as follows:

(1) $$\bar{X}=\frac{\sum {\bar{x}}_{i}}{N}$$

where ${\bar{x}}_{i}$ is the maximum, mean, minimum value for each individual patient, and $\bar{x}$ is the final maximum, mean, minimum value for the whole group, and N is the total number of the patients. The standard deviation Δ was also calculated through the whole group as:

(2) $$\Delta =\sqrt{\frac{\sum \delta_{i}^{2}}{N}}$$

where Δ is the group standard deviation, $\delta_{1}$ is the standard deviation for each patient, and *N* is the total number of the patients.

The results were reported according to the ExacTrac coordinate system: with the patient supine position and head toward the gantry, the patient inferior–superior is the positive longitudinal direction, the patient right–left is the positive lateral direction, and the positive vertical direction is pointing from the patient posterior to anterior. The rotation direction is along the 3D shifting axis. For example, the lateral rotation is along positive lateral axis, following right hand rule.

## III. RESULTS

**Figure 1:**
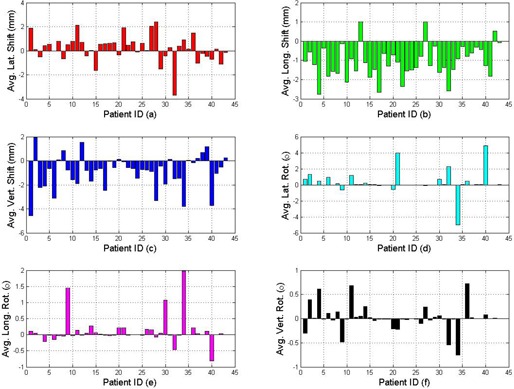
Average shifts in 6D for different patients: (a) lateral shifts; (b) longitudinal shifts; (c) vertical shifts; (d) rotation angle around lateral axis; (e) rotation angle around longitudinal axis; (f) rotation angle around vertical axis.

**Figure 2:**
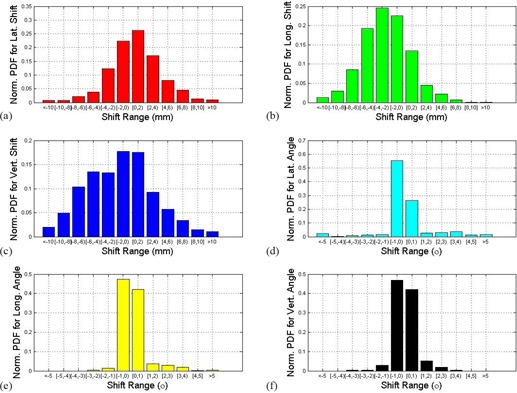
Normalized PDF plots for interfraction motion in 6D (a–f from top to bottom and from left to right): (a) lateral shifts; (b) longitudinal shifts; (c) vertical shifts; (d) rotation angle around lateral axis; (e) rotation angle around longitudinal axis; (f) rotation angle around vertical axis.

Figure 1 shows the mean shifts of all the treatment fractions (both interfraction and intrafraction shifts) for each individual patient in 6D. More patients have the potential to shift positive lateral, negative longitudinal, negative vertical direction according to the mean results. Small angle rotations were observed most of the time. However, large angle rotation along lateral direction did happen. The reasons may mainly be due to patient anatomy change from fraction to fraction and/or the therapist's manual setup error according to the tattoo makers. Figure 2 shows the normalized PDF plots of all the patients of all the fractions in 6D shifts. The x‐axis represents the shift range. The y‐axis is the normalized PDF for the corresponding range. The minimum, average, and maximum values with standard deviation are listed in Table 1. Here we reported the results with signs, and the signs represent directions. We do so in order to provide visualization of the direction for the shifts. The whole group's PDFs show that the patient shifts have a shape similar to Gaussian distribution in translation shifts. A slight preference to positive lateral direction, negative longitudinal direction, and vertical direction is observed in Fig. 1. Most of the rotation shifts are within ± 2° range.

**Table 1:** Interfraction 6D shifts.

	*Minimum*		*Average*		*Maximum*	
	*Value*	±SD	*Value*	±SD	*Value*	±SD
Lat. Shift (mm)	−5.12	3.89	0.20	2.21	6.07	4.44
Long. Shift (mm)	−6.80	3.21	−1.09	2.21	3.12	2.62
Vert. Shift (mm)	−7.33	3.46	−0.93	2.70	5.93	4.85
Lat. Rot. (°)	−1.23	1.95	0.25	1.30	2.38	2.91
Long. Rot (°)	−0.67	0.91	0.10	0.61	1.51	2.04
Vert. Rot (°)	−0.75	1.01	0.02	0.50	0.82	1.13

**Figure 3:**
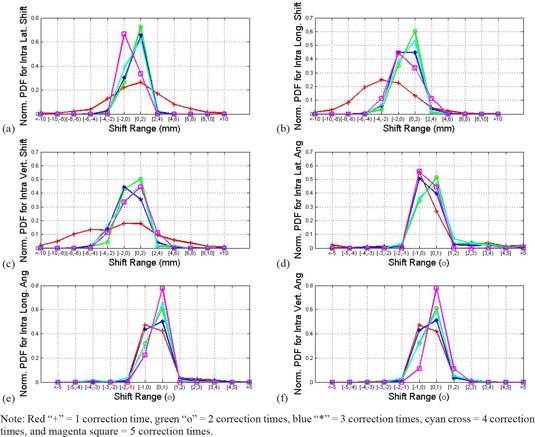
Normalized PDF plots for intrafraction motion in 6D (a–f from top to bottom and from left to right): (a) lateral shifts; (b) longitudinal shifts; (c) vertical shifts; (d) rotation angle around lateral axis; (e) rotation angle around longitudinal axis; (f) rotation angle around vertical axis. Note: Red “+”=1 correction time, green “+”=2 correction times, blue “*”=3 correction times, cyan cross=4 correction times, and magenta square=5 correction times.

Figure 3 illustrates normalized PDFs in 6D for intrafraction motion for all the patients. Each curve represents correction times from 1 to 5. It shows clearly the shifts overall were reduced by corrections in all 6D in three translation directions with the PDF narrow distribution when comparing the other curves to the red one.

**Figure 4:**
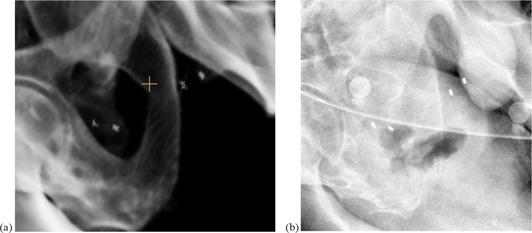
Implanted markers and isocenter (a) and the verification image (b). The arrows point to the four markers.

Figure 4(a) shows the digitally reconstructed radiograph (DRR) with isocenter and the corresponding setup X‐ray image (Fig. 4(b)). The marks are clearly shown on the X‐ray image and easily to identify for marker matching. The average four markers registration uncertainties (recorded marker position vs. planned marker position) are: 3.39±3.19 mm, 3.35±3.20 mm, 3.35±3.18 mm, and 3.29±3.19 mm for marker #1 to #4, respectively. The registration uncertainty caused the center of mass of the four markers to have averaged registration shift of 3.14±1.23 mm.

**Figure 5:**
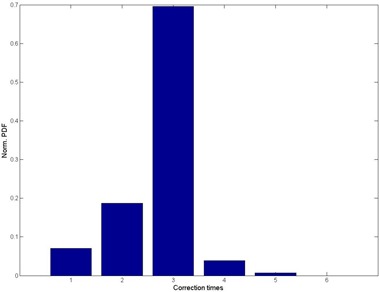
The correction times vs. normalized probability density function (PDF) plot for prostate setup marker matching for all the patients. The plot has been normalized to 1.

The correction times PDF is illustrated in Fig. 5. On average, each patient had three correction times per fraction. Imaging dose for the corrections may be a concern. Linthout et al.^(^ ^11^ ^)^ have done a study showing that the radiation dose per X‐ray image using the ExacTrac system is 0.5 mSv per X‐ray exposure. Three correction times will result in a radiation dose of about 3 mSv per X‐ray exposure. Here the 3 mSv is total dose to the patient. The dose is still less than kilovoltage cone‐beam computed tomography (kV‐CBCT) imaging dose, which is in the range of several cGy or tens of cGy.^(^ ^12^ ^)^

With a tolerance of 1 mm for isocenter intrafraction shifts and marker matching setup, the prostate IMRT treatment can achieve less than 2 mm shifts in 3D and less than 0.25° rotation adjustments on average, based on Table 1 average results. The results can help the prostate IMRT treatment achieve better setup accuracy and precision. Alongso‐Arrizabalaga et al.^(^ ^13^ ^)^ found similar results in 3D shift. The results may help the physician reduce the PTV margin^(^ ^13^ ^)^ for the prostate patient with markers implanted and treated with ExacTrac 6D system.

## IV. DISCUSSION

The interfraction setup uncertainties are the major patient setup uncertainty sources. With marker matching, it can be reduced. Similar conclusion was found by Alongso‐Arrizabalaga et al.^(^ ^13^ ^)^ The markers matching process may have a few clinical issues, such as marker migrating, bent coil, and symmetric pattern for seeds in which ExacTrac system cannot identify the rotation, etc. However, it works well overall.

The intrafraction motion can be well‐controlled with snapshot mode. If the motion is out of tolerance, adjustment is necessary. The uncertainty will be further reduced with more monitoring time and adjustments. However, frequent adjustments may not be necessary and will cost more treatment time.

Rotation angles in 3D are small and may not need to be adjusted. The results shown were calculated by the ExacTrac system. We did not apply the rotation shifts. However, if the rotation shift angles were larger than 2°, further investigation was needed and the patient was repositioned.

The visibility of the either gold seeds or Visicoil was good for the kV X‐ray images. As shown in the Fig. 4(b), the markers are easy to be identified by the users. The gold seeds may migrate; in that case, the three seeds instead of four seeds matching will be used. The Visicoil is not easy to migrate. In some cases, the Visicoil may be bent at one end; therefore, three marker matching is recommended instead of four.

It seems that a frequency of three for the correction times is good. The more times the PTV motion is monitored, the better are the results. However, the user has to balance the treatment time, imaging dose, and the adjustment times.

## V. CONCLUSIONS

In this study, we have done the analysis of the prostate patient setup uncertainties using ExacTrac 6D X‐ray systems. Our results showed that for a patient with markers implanted, we can achieve fewer than 2 mm setup shifts on average in 3D translations, and less than 0.25° rotation as overall interfraction mean error. The imaging dose is less than kV (CBCT) for setup verification. A tighter margin for the planning target volume (PTV) may be applied if the prostate patient has markers implanted in addition to using ExacTrac 6D X‐ray system for setup.
